# Procrastination Among University Students: Differentiating Severe Cases in Need of Support From Less Severe Cases

**DOI:** 10.3389/fpsyg.2022.783570

**Published:** 2022-03-15

**Authors:** Alexander Rozental, David Forsström, Ayah Hussoon, Katrin B. Klingsieck

**Affiliations:** ^1^Department of Psychology, Uppsala University, Uppsala, Sweden; ^2^Department of Clinical Neuroscience, Karolinska Institutet, Stockholm, Sweden; ^3^Great Ormond Street Institute of Child Health, University College London, London, United Kingdom; ^4^Department of Psychology, Stockholm University, Stockholm, Sweden; ^5^Department of Psychology, Faculty of Arts and Humanities, Paderborn University, Paderborn, Germany

**Keywords:** procrastination, students, assessment, severity, differentiation

## Abstract

Procrastination refers to voluntarily postponing an intended course of action despite expecting to be worse off for this delay, and students are considered to be especially negatively affected. According to estimates in the literature, at least half of the students believe procrastination impacts their academic achievements and well-being. As of yet, evidence-based ideas on how to differentiate severe from less severe cases of procrastination in this population do not exist, but are important in order to identify those students in need of support. The current study recruited participants from different universities in Sweden to participate in an anonymous online survey investigating self-rated levels of procrastination, impulsivity, perfectionism, anxiety, depression, stress, and quality of life. Furthermore, diagnostic criteria for pathological delay (PDC) as well as self-report items and open-ended questions were used to determine the severity of their procrastination and its associated physical and psychological issues. In total, 732 participants completed the survey. A median-split on the Pure Procrastination Scale (PPS) and the responses to the PDC were used to differentiate two groups; “less severe procrastination” (PPS ≤ 2.99; *n* = 344; 67.7% female; *M* age = 30.03; *SD* age = 9.35), and “severe procrastination” (PPS ≥ 3.00; *n* = 388; 66.2% female; *M* age = 27.76; *SD* age = 7.08). For participants in the severe group, 96–97% considered procrastination to a problem, compared to 42–48% in the less severe group. The two groups also differed with regard to considering seeking help for procrastination, 35–38% compared to 5–7%. Participants in the severe group also reported more problems of procrastination in different life domains, greater symptoms of psychological issues, and lower quality of life. A thematic analysis of the responses on what physical issues were related to procrastination revealed that these were characterized by stress and anxiety, e.g., tension, pain, and sleep and rest, while the psychological issues were related to stress and anxiety, but also depression, e.g., self-criticism, remorse, and self-esteem. The current study recommends the PPS to be used as an initial screening tool, while the PDC can more accurately determine the severity level of procrastination for a specific individual.

## Introduction

In academia, procrastination is a well-known, almost commonplace phenomenon. Students often delay tasks and activities inherent to learning and studying, despite knowing that they will be worse off because of the delay (cf. Steel, 2007; Steel and Klingsieck, 2016). For some students, academic procrastination can be specific to a situation (i.e., state procrastination), for others it takes on features of a habit or a disposition (i.e., trait procrastination). Studies estimate that almost all students engage in procrastination once in a while, while 75% consider themselves habitual procrastinators (Steel, 2007). For almost half of these habitual procrastinators, procrastination is a real and persistent problem (Steel, 2007), and something they would like to tackle (Grunschel and Schopenhauer, 2015). It can be assumed, however, that not all of them seek help due to the self-regulative problems inherent to procrastination, and, even more so, due to feelings of shame associated with procrastination (Giguère et al., 2016).

In light of the negative consequences, procrastination can have for academic achievement (e.g., Kim and Seo, 2015), and well-being (cf. Sirios and Pychyl, 2016), it seems important to screen for cases of severe procrastination in a student population in order to offer the support needed. In the case of students who do seek help in student health centers, it is also helpful to see whether they represent a case of severe or less severe procrastination so that support can be tailored to their specific needs.

The aim of the current study is, thus, to differentiate between students who might be in need of professional help from those with less pressing concerns. This is done by determining what characterizes severe and less severe procrastinators with regard to their level of anxiety, depression, stress, quality of life, impulsivity, perfectionism, and demographic variables. Procrastination itself is also assessed by two different self-report measures with the intention of proposing ways of screening in a student population. This could help therapists identify those in need of guidance so that effective interventions can be introduced. For college and university students this would be particularly useful as they find themselves in a setting where procrastination is particularly endemic, often lack the necessary resources or strategies to overcome problems on their own, and procrastination can have dire consequences not only for their academic achievements but also physical and psychological well-being.

## Conceptual Framework

### Academic Procrastination

The prominent definition of procrastination as “to voluntarily delay an intended course of action despite expecting to be worse off for the delay” (Steel, 2007, p. 66) reflects two important aspects of the phenomenon. First, procrastination is a post-decisional phenomenon in goal-directed behavior in that an intention (e.g., to study for an exam) has been formed. Second, procrastination is acratic in nature since individuals put of the intended course of action contrary to knowing better. This acratic nature is reflected by feelings such as regret, shame, guilt, worry, and anxiety (e.g., Giguère et al., 2016). It is important to acknowledge that a delay is not procrastination if it is strategic or results from causes not under the control of the individual (cf. Klingsieck, 2013). Taking these aspects – post-decisional, acratic, and non-strategic – together, suggests that procrastination is a failure in self-regulation (cf. Steel, 2007), This is the most popular conceptualization of procrastination in the literature. In fact, the dispositional, the motivational-volitional, the clinical, and the situational perspective on procrastination can be boiled down to this understanding of procrastination (Klingsieck, 2013). As for students, while academic procrastination is just a little nuisance for some, it entails serious problems for others.

### Procrastination’s Link to Depression, Anxiety, Stress, and Quality of Life

Procrastination is associated with negative consequences concerning performance as well as physical and psychological well-being. However, although never a particularly helpful behavior, the relationship with performance is probably not as strong as most would expect. Among students, the correlation with academic achievement is weak, *r*s = –0.13 to –0.19 (Steel, 2007; Kim and Seo, 2015), and perhaps not the main reason for why individuals regard procrastination as a problem. Instead, it might be its effects on physical and psychological well-being that eventually makes someone seek professional help (Rozental and Carlbring, 2014). In a qualitative study of 36 students, for instance, the most frequently reported negative consequences were anger, anxiety, feelings of discomfort, shame, sadness, feeling remorse, mental stress, and negative self-concept (Grunschel et al., 2013). Systematic reviews and meta-analyses on the link between procrastination and symptoms of psychiatric conditions have also found a weak but nonetheless clinically meaningful correlation with depression, *r*s = 0.28 to 0.30 (van Eerde, 2003; Steel, 2007). The same also goes for anxiety, *r* = 0.22 (van Eerde, 2003). Studies investigating the connection between self-report measures in different populations have demonstrated stronger correlations, such as Rozental et al. (2015) in a clinical trial of adults seeking treatment for procrastination (*n* = 710), *r* = 0.35 for depression and *r* = 0.42 for anxiety. Similar results were also obtained by Beutel et al. (2016) in an adult community sample (*n* = 2527), *r* = 0.36 for depression and *r* = 0.32 for anxiety. Although both lower mood and increased unrest can, in themselves, cause procrastination, it is assumed that procrastination also creates a downward spiral characterized by negative thoughts and feelings (Rozental and Carlbring, 2014).

Apart from depression and anxiety, students generally tend to regard procrastination as something stressful. Stead et al. (2010) investigated this association using self-report measures in a sample of students (*n* = 200), demonstrating a weak but nonetheless significant correlation between procrastination and stress, *r* = 0.20. Similar findings were reported by Sirois et al. (2003) for students (*n* = 122), and Sirois (2007) for a sample of community-dwelling adults (*n* = 254), *r*s = 0.13 to 0.20. Further, Beutel et al. (2016) found somewhat stronger correlations with stress, *r* = 0.39, as well as with burnout, *r* = 0.27. Stress might also play a role as mediator between procrastination and illness, as proposed by the so-called procrastination-health model by Sirois (2007), implying that procrastination not only leads to more stress, but that the increase in stress in turn leads to many physical issues. Meanwhile, in terms of quality of life and satisfaction with life, procrastination exhibits a weak negative correlation, *r* = −0.32 (Rozental et al., 2014), and *r* = −0.35 (Beutel et al., 2016), meaning that procrastination could take its toll on how one appreciates current circumstances.

However, despite the fact that procrastination might be affecting physical and psychological well-being negatively, it is still unclear when it goes from being a more routine form of postponement to becoming something that warrants support, for instance in the realm of counseling or therapy. The literature suggests that as many as 20% of the adult population could be regarded as “chronic procrastinators” (Harriot and Ferrari, 1996, p. 611), a number that is easily surpassed by the 32% of students that were characterized as “severe, general procrastinators” (Day et al., 2000, p. 126). Students are generally considered worse-off when it comes to recurrently and problematically delaying important curricular activities, with more than half of this population stating that they would like to reduce their procrastination (Solomon and Rothblum, 1984). Still, all of these rates rely on arbitrary cutoffs on specific self-report measures, such as exceeding a certain score, or do not define what is meant by procrastination, which may not correspond to something that requires clinical attention (Rozental and Carlbring, 2014). Establishing a more valid cutoff is therefore needed in order to separate the less severe cases of procrastination from those having problems to the degree that it severely affects everyday life.

### Procrastination’s Link to Impulsivity and Perfectionism

Two other variables that are frequently explored in relation to procrastination involve impulsivity and perfectionism. These might be especially pertinent to examine in the context of students who, due to their age, are more impulsive and engage in more reckless behaviors, such as binge drinking (Lannoy et al., 2017), but also tend to perceive the relentless pursuit of high standards as socially desirable despite the fact it can become maladaptive (Stoeber and Hotham, 2013). Research has found that impulsivity is moderately correlated with procrastination, *r* = 0.41 (Steel, 2007), making it one of the strongest predictors among the personality traits. A twin study by Gustavson et al. (2014) confirmed this association (*n* = 663), suggesting that the genetic correlation between impulsivity and procrastination is perfect, *r* = 1.0. However, this was later questioned by a twin study with a much larger sample (*n* = 2012), demonstrating a weak but nonetheless noteworthy correlation, *r* = 0.29 (Loehlin and Martin, 2014). Rozental et al. (2014) also examined the link between impulsivity and procrastination, but using a self-report measure of susceptibility to temptation, indicating a moderate correlation, *r* = 0.53. At its core, impulsivity shares many features with procrastination (i.e., self-regulatory failure), making it reasonable to expect a strong connection between the two constructs. Meanwhile, the relationship between perfectionism and procrastination has been disputed. Originally, Steel (2007) demonstrated a non-significant correlation, *r* = −0.03. Similarly, the correlation by van Eerde (2003) was weak, *r* = 0.12. This goes against the clinical impression by many therapists that perfectionism often leads to procrastination. However, in both of these cases perfectionism was perceived as a unidimensional construct. There is currently consensus that perfectionism in fact has two higher-order dimensions; (1) perfectionistic strivings, i.e., setting high standards and expecting no less than perfection from yourself, and (2) perfectionistic concerns, i.e., being highly self-critical and overly concerned about others’ perception of you, and having a hard time enjoying your achievements. A recent systematic review and meta-analysis separating these two demonstrated a more complex relationship with procrastination (Sirois et al., 2017). Perfectionistic strivings had a weak negative correlation with procrastination, *r* = −0.22, while perfectionistic concerns had a weak positive correlation with procrastination, *r* = 0.23. In other words, setting and striving for high standards might actually be associated with less procrastination, while the more neurotic aspects of perfectionism are related to more procrastination.

To what extent impulsivity and perfectionism might differ between cases of less severe and severe cases of procrastination is currently unknown. However, just as physical and psychological well-being is expected to be more negatively affected among those who exhibit higher levels of procrastination, impulsivity and perfectionism should be more pronounced.

### The Current Study

The aim of the current study is to investigate all of these aspects in a sample of students with the purpose of trying to differentiate between those who might be in need for professional help from those with less pressing matters. The idea is to outline their respective characteristics with regard to scores on self-report measures on anxiety, depression, stress, quality of life, impulsivity, and perfectionism, and demographics. Procrastination itself is assessed by two different self-report measures. This first measure is the Pure Procrastination Scale (PPS; Steel, 2010) which is a widely used self-report measure. The second measure are the recently proposed diagnostic criteria for pathological delay (Pathological Delay Criteria; PDC; Höcker et al., 2017).

The second aim of the current study is to explore the physical and psychological issues related to procrastination on a deeper level. This is made possible through a qualitatively analysis of the responses to two open-ended questions regarding the impact of recurrently putting off activities that need to be completed. Prior research has by qualitative means primarily studied the antecedents of procrastination (Klingsieck et al., 2013), but rarely its implications for physical and psychological well-being. One notable exception is the interview study by Grunschel et al. (2013) cited in the introduction. Investigating these experiences in detail and how often they occur could provide a better understanding of how procrastination affects someone physically and psychologically, and in turn when further assistance might be necessary.

## Materials and Methods

### Procedure

The study received ethical approval from the Swedish Ethical Review Authority in June 2020 (Dnr: 2020-00555). Advertisements for the study were initially sent out in October 2020 via the communications office of Karolinska Institutet, which is a medical university in Stockholm, Sweden. However, in order to recruit students from other backgrounds, information about the study was also forwarded to two additional universities in Sweden and posted on various student forums on Facebook, LinkedIn, Accindi, and Instagram. Using a link to a website created specifically for the study, the student could then read about the research aims and design, procedures for data collection and management, ethics, and the principal investigator. The student was also informed that a 45-min pre-recorded lecture with the first author on procrastination would follow once the survey was completed, as a small token of gratitude for the student’s participation. After submitting informed consent, the student was forwarded to an anonymous survey managed through Limesurvey. Both, the website and the survey itself, were available in Swedish and English. The whole survey took on average 21 min for the participants to complete (*SD* = 16 min), and always followed the same order of presentation, i.e., no randomization of self-report measures or items were made. Every item of a self-report measure had to be completed to progress to the next, presenting only one self-report measure per page and using a progress bar on top of the screen to convey how much was left on the survey.

### Sample

In total, 806 students decided to open the link and 797 actually started filling out the survey, resulting in 732 complete survey responses (90.8%). There were no systematic differences between completers and non-completers concerning their demographic information and procrastination, with the exception of civil status (see Appendix for the specifics). Of those who finished the survey, 66.6% were female, which corresponds with the most recent numbers on the gender distribution of newly admitted university students in Sweden (58% female; Swedish Higher Education Authority, 2020). The mean age was 28.8 years (*SD* = 8.30; range 18–65). They were either single (44%) or married (54%), and the vast majority had no children (78%). In terms of their education, 6.8% attended just a single course, (e.g., Nutrition, the nutrients, and metabolism, 7.5 higher education credits), 63.7% underwent a complete study program, such as the study program in dental hygiene (180 higher education credits), 9.1% were enrolled in post graduate studies, for example the study program in psychotherapy (90 higher education credits), and 3.4% were admitted as doctoral candidates. Of note, 30 higher education credits correspond to one semester full-time. The participants had, on average, achieved 195 higher education credits (*SD* = 141), which thus corresponds to 3.25 years of full-time education. With regard to psychiatric disorders, 115 self-reported having a diagnosis (15.7%). These were grouped according to the responses to an open-ended question, with mixed conditions representing the largest category (40%, i.e., having more than one diagnosis, mostly a combination of depression and anxiety), followed by depression (13.9%), and ADHD (13%). As for questions regarding procrastination, 71% considered it to be a problem, with a mean age of 17.5 years (*SD* = 5.7; range 10–53) for when they first started perceiving it as problematic, and 29.4% of this group had considered seeking help for procrastination. None of these variables differed between genders, see Table 1 for an overview.

**TABLE 1:** Descriptive statistics for whole sample and results of *t*-tests of gender differences.

		Whole sample (*N* = 732)				Female (*n* = 488)		Male (*n* = 241)					
		*M*	*SD*	*Min*	*Max*	*M*	*SD*	*M*	*SD*	*t*	*df*	*p*	*d*
Age		28.83	8.30	18	65	29.40	8.89	27.72	6.85	2.80	600.54	0.01	0.20
Education Credits		194.99	140.85			196.34	137.67	192.00	146.44	0.39	723.00	0.70	0.03
Age Start Procrastination		17.53	5.74	10	53	17.79	5.98	17.11	5.29	1.25	486.00	0.21	0.12
Pure Procrastination Scale (PPS)		3.00	0.91	1.00	5.00	2.96	0.92	3.07	0.89	1.50	727	0.13	0.12
**Effects of Procrastination on Life-Domains**													
	Interests/leisure	4.68	2.940	0.00	10.00	4.90	2.90	4.25	2.99	2.41	512	0.02	0.22
	Work/studies	7.76	1.923	0.00	10.00	7.75	1.95	7.78	1.89	2.41	512	0.02	0.02
	Friendships/social life	4.50	2.801	0.00	10.00	4.57	2.72	4.38	2.95	0.76	512	0.45	0.07
	Community/engagement/spirituality	3.09	3.192	0.00	10.00	3.21	3.24	2.86	3.11	1.16	512	0.25	0.11
	Family life/parenting	3.71	2.810	0.00	10.00	3.91	2.84	3.39	2.74	1.99	512	0.05	0.18
	Rest/sleep	6.36	2.992	0.00	10.00	6.50	3.00	6.04	2.96	1.67	512	0.10	0.15
	Love/intimate relationships	3.79	3.179	0.00	10.00	3.71	3.07	3.97	3.37	0.86	347.223	0.39	0.08
	Physical activity/diet	6.02	2.930	0.00	10.00	6.33	2.84	5.43	3.01	3.37	512	0.00*	0.31
Susceptibility to Temptation Scale (STTS)		3.15	0.93	1.00	5.00	3.11	0.94	3.24	0.91	1.78	727	0.08	0.14
**Clinical Perfectionism Questionnaire (CPQ)**													
	CPQ_Personal Standards	2.66	0.63	1.00	4.00	2.74	0.60	2.49	0.65	5.01	441.77	0.00*	0.41
	CPQ_Emotional Concerns	2.93	0.76	1.00	4.00	3.00	0.72	2.78	0.81	3.61	429.22	0.00*	0.30
Generalized Anxiety Disorder Questionnaire (GAD-7)		7.85	5.59	0.00	21.00	8.36	5.72	6.79	5.20	3.71	521.32	0.00*	0.28
Patient Health Questionnaire (PHQ-9)		8.95	6.34	0.00	27.00	9.12	6.35	8.54	6.24	1.16	727	0.25	0.09
Perceived Stress Scale (PSS)		2.91	0.65	1.14	4.71	2.78	0.59	2.66	0.61	2.61	727	0.01	0.21
**Brunnsviken Brief Quality of Life Scale (BBQ)**													
	BBQ_Leisure time	7.87	4.81	0.00	16.00	7.87	4.76	7.88	4.92	–0.03	727	0.98	0.02
	BBQ_View of Life	9.68	5.14	0.00	16.00	9.88	5.08	9.32	5.22	1.39	727	0.17	0.11
	BBQ_Creativity	6.83	5.12	0.00	16.00	6.91	5.14	6.60	5.04	0.77	727	0.44	0.06
	BBQ_Learning	9.61	5.03	0.00	16.00	9.83	4.99	9.20	5.11	1.61	727	0.11	0.13
	BBQ_Friends	9.76	5.32	0.00	16.00	10.06	5.35	9.15	5.23	2.16	727	0.03	0.17
	BBQ_Self	8.70	5.04	0.00	16.00	8.86	4.99	8.44	5.11	1.07	727	0.29	0.08
	BBQ_Total	52.46	21.21	0.00	96.00	53.41	21.01	50.58	21.62	1.68	466.01	0.09	0.13

*p < 0.002 (Bonferroni correction); Calculations based on sum scores for GAD-7, PHQ-9, and BBQ.

### Instruments

#### Procrastination

In order to differentiate and classify the more severe cases of procrastination, a widely used self-report measure is applied, the Pure Procrastination Scale (PPS), which was originally introduced and validated by Steel (2010), and translated to a large number of languages since (Svartdal et al., 2016). The PPS was developed from several other self-report measures, retaining only those items that demonstrated the strongest factor loadings on the core construct of procrastination (i.e., not other forms of delay), hence the name “pure.” The PPS has 12 items, e.g., “I often find myself performing tasks that I had intended to do days before” (item 6), is scored according to a 5-point Likert-scale (1–5), and has an internal consistency in the current study of Cronbach’s α = 0.92.

Secondly, diagnostic criteria for pathological delay (Pathological Delay Criteria; PDC), which were put forward in a therapy manual by Höcker et al. (2017), are also used to differentiate between less and more severe cases of procrastination. According to the PDC, procrastination can be considered pathological if the following two criteria are met:

Over the past 6 months…

(1) On at least half of the days, important tasks were delayed past the adequate point in time, even though there was sufficient time to complete them.
(2) Procrastination has strongly interfered with reaching personally relevant goals.

In addition, at least three of following criteria also need to be fulfilled:

(1) More than half of the time available for completing a task was wasted by procrastinating.
(2) On at least half of the days, other less important tasks were preferred, even though the individual wanted to start working on the more pressing tasks.
(3) On at least half of the days, the delay caused aversion and animosity.
(4) At least half of the tasks that were to be completed were finished only under great time pressure or not at all due to procrastination.
(5) At least half of the individual’s performance potential was impaired due to procrastination.
(6) The individual has experienced physical issues due to procrastination (e.g., tensed muscles, sleeping disorders, cardiovascular problems, gastric, and digestive problems), or psychological issues due to procrastination (e.g., restlessness, feeling of being pressured, feeling of being helpless, inner tension, and anxiety).*

* At least five of these issues need to be reported to meet this criterium.

The criteria above were developed as a diagnostic instrument for differential diagnosis and as a basis for clinical decision making. During its development, the authors followed the definition and structure of psychiatric disorders used by the Diagnostic and Statistical Manual of Mental Disorders (American Psychiatric Association, 2013). In order to select the criteria with the best predictive value, large samples of university students seeking help at a procrastination clinic at the University of Münster, Germany, were used (e.g., Engberding et al., 2011). The authors used the methods of best subset regression and ROC-analyses to select the criteria with the highest scores on sensitivity and specificity for identifying pathological delay. These criteria and the corresponding questionnaire were subsequently published in the therapist manual (Höcker et al., 2017).

Further variables of meaningful aspects concerning procrastination were assessed: (1) if the participant itself believes procrastination is a problem and, if yes, (2) at what age the participant started perceiving procrastination as a problem, (3) if the participant has ever considered seeking help for procrastination, and (4) the impact of procrastination on various life domains. In order to assess how procrastination had affected the participants, its negative effects on eight different life domains were probed for: “To what degree do you think procrastination has affected you negatively in the following life domains?”. The life domains were: interest/leisure, work/studies, friendships/social life, community/engagement/spirituality, family life/parenting, rest/sleep, love/intimate relationships, and physical activity/diet. Participants rated each life domain using a 10-point Likert-scale ranging from 0 = not at all to 10 = very much. The life domains were inspired by the type of value measures often used in Acceptance and Commitment Therapy (Reilly et al., 2019), and are commonly employed in many clinical trials (e.g., Buhrman et al., 2020; Ehlers et al., 2020).

#### Impulsivity

Impulsivity was assessed using the Susceptibility to Temptation Scale (STS; Steel, 2010; Svartdal et al., 2016), which is comprised of 11 items regarding the inclination to fall for more immediate gratifications, e.g., “I will crave a pleasurable diversion so sharply that I find it increasingly hard to stay on track” (item 1). The STS is scored on a 5-point Likert-scale (1–5), and has an internal consistency in the current study of α = 0.93.

#### Perfectionism

Perfectionism was assessed by the Clinical Perfectionism Questionnaire (Dickie et al., 2012). This scale assesses the frequency of dysfunctional self-imposed standards in the last 4 weeks by a subscale covering the personal standards (i.e., perfectionistic standards), and a second subscale covering emotional concerns and consequences (i.e., perfectionistic concerns). Item 9 of the original scale (“Have you repeatedly checked how well you are doing at meeting your standards [for example, by comparing your performance with that of others]?”) was omitted because it did not load on the factor perfectionistic standards as in the original version by the authors. Item 2 of the subscale perfectionistic concerns (“Have you tended to focus on what you have achieved, rather than on what you have not achieved?”) was omitted due to a very low item-scale-correlation. Thus, the subscale Personal Standards (CPQ_PS) was composed of five items (α in current study = 0.71; sample item “Have you been told that your standards are too high?”). The subscale Emotional Concerns (CPQ_EC) was composed of three items (α in current study = 0.76; sample item “Have you been afraid that you might not reach your standards?”). The CPQ is scored on a four-point Likert-scale (1–4).

#### Anxiety

Anxiety was examined using the Generalized Anxiety Disorder – 7 Items (GAD-7; Spitzer et al., 2006). It consists of seven items concerning the general level of anxiety and worry experienced during the last 2 weeks, and is often used as a screening tool for anxiety disorders, e.g., “Over the last 2 weeks, how often have you been bothered by the following problems: Worrying too much about different thing” (item 3). The GAD-7 is scored on a four-point Likert-scale (0–3), and has an internal consistency in the current study of α = 0.90. A score of 5 points indicate mild anxiety, 10 moderate anxiety, and 15 severe anxiety.

#### Depression

Depression was assessed by the Patient Health Questionnaire – 9 Items (PHQ-9; Kroenke et al., 2001). It has nine items on depressive symptoms experienced during the last 2 weeks, in accordance with the diagnostic criteria for major depressive disorder (American Psychiatric Association, 2013), e.g., “Over the last 2 weeks, how often have you been bothered by any of the following problems? Little interest or pleasure in doing things” (item 1). The PHQ-9 is scored on a four-point Likert-scale (0–3), and has an internal consistency in the current study of α = 0.88. A score of 5 points indicate mild depression, 10 moderate depression, 15 moderately severe depression, and 20 severe depression.

#### Stress

Stress was explored using the Perceived Stress Scale (PSS; Cohen et al., 1983). It is comprised of 14 items regarding stress in different situations, as experienced during the last month, e.g., “In the last month, how often have you felt that you were unable to control important things in your life?” (Item 2). The PSS is scored on a five-point Likert-scale (1–5), and has an internal consistency in the current study of α = 0.85.

#### Quality of Life

Quality of life was determined by the Brunnsviken Brief Quality of Life Scale (BBQ; Lindner et al., 2016). It features six life domains (leisure time, view of one’s own life, learning, creativity, friends and friendship, yourself as a person), and is rated on both importance and how satisfied one is with each domain, e.g., “I am satisfied with my leisure time; I have the opportunity to do what I want in order to relax and enjoy myself.” (domain 1). The BBQ is scored on a 5-point Likert-scale (0–4), where importance and satisfaction in each domain are multiplied and summing the products for a total score (range 0–96). These weighted ratings as well as the total score for quality of life was used for the current study. The BBQ has an internal consistency of α = 0.79 in the current study.

In addition, achieved higher education credits was assessed to differentiate the two groups by their academic achievement. Age and gender were assessed as demographic variables but only used to characterize the sample and not to differentiate the groups.

### Quantitative Analysis

Multiple *t*-tests and Chi^2^-tests were performed by SPSS Version 27. The significance level was corrected (Bonferroni) to *p* < 0.002 (*t*-tests) and 0.007 (Chi^2^-Tests). In order to differentiate severe cases from less severe cases of procrastination, the sample was split along the median (*Med.* = 3.00) of the PPS. This created two groups, which are referred to as: “less severe procrastination” (PPS ≤ 2.99; *n* = 344; 67.7% female; *M*_*age*_ = 30.03; *SD_*age*_* = 9.35), and “severe procrastination” (PPS ≥ 3.00; *n* = 388; 66.2% female; *M*_*age*_ = 27.76; *SD*_*age*_ = 7.08). For the second differentiation, the PDC was used to split the sample into the corresponding groups (i.e., based on whether the participants fulfilled all of the necessary criteria or not): “less severe procrastination” (*n* = 398; 71.5% female; *M*_*age*_ = 29.94; *SD*_*age*_ = 9.03), and “severe procrastination” (*n* = 344; 61.6% female; *M*_*age*_ = 27.51; *SD_*age*_* = 7.11).

### Qualitative Analysis

Two items of the PDC were open-ended and therefore analyzed qualitatively. Given the nature of these variables and their manifest content, that is, being short text-based survey responses with little room for elaboration, inductive thematic analysis was deemed appropriate to use. Inductive refers to generating a new understanding of the subject matter, rather than testing a predefined theoretical framework during the analysis (Thomas, 2006). Meanwhile, thematic analysis is a procedure for qualitative analysis considered suitable for exploring recurrent patterns or themes within data. Braun and Clark (2006) provide an overview of the steps in the analytic process, which usually includes familiarizing yourself with your data by reading it repeatedly and taking notes, extracting meaningful entities of relevance to the purpose of the study, generating codes representing important issues for further inquiry, collating the codes to explore potential themes, reviewing the themes by going back and forward to your data, naming the themes, and reporting and discussing the results. The first author conducted the thematic analysis and discussed the results with the last author, but no further attempt at cross-validation was considered necessary given the characteristics of the data. The first author is a Swedish clinical psychologist and researcher with extensive experience of treating and researching procrastination, perfectionism, anxiety disorders, and exhaustion disorder, and has worked with both quantitative and qualitative methods.

The first qualitative item of the PDC concerned the physical issues of procrastination and involved a dataset of 2304 words (the average number of characters per response was 59.8, *SD* = 92.7). The second qualitative item of the PDC concerned the psychological issues of procrastination and was comprised of 4022 words (the average number of characters per response was 55.8, *SD* = 67.5). Because of a high degree of overlap in the responses, such as a vast majority reporting experiencing anxiety regardless of being a severe procrastinator or not, and that each response could entail a large number of physical as well as psychological issues, the variables could only be analyzed and presented qualitatively, rather than being part of the quantitative analysis.

## Results

### Descriptive Statistics

Table 1 presents the descriptive statistics for each self-report measure as well as their respective gender differences (female vs. male). There were only statistically significant gender differences on the CPQ (Cohen’s *d* = 0.30 and 0.41), and GAD-7 (*d* = 0.28), with female students scoring higher than male students. As for procrastination, the average score was 3.00 (*SD* = 0.91), which is the same as the median split used for grouping the participants into severe and less severe procrastinators, while 46% of the sample fulfilled the PDC criteria. Negative effects of procrastination were most prominent in the life domains of work/studies, physical activity/diet, and rest/sleep, and being considerably lower in the life domains of family life/parenting and community/engagement/spirituality. The average scores on the GAD-7 and PHQ-9 correspond to mild anxiety and mild depression.

### Differentiating Severe Cases From Less Severe Cases of Procrastination

The results of differentiating severe cases from less severe cases of procrastination are presented in detail in Tables 2–4. The two groups diverged with regard to their perception of procrastination. In the group “severe procrastination,” almost every participant (96–97%) considered procrastination to be a problem, while those participants belonging to the group “less severe procrastination” did so to a much lesser extent (42–48%). In addition, 35–38% of the severe procrastinators had considered seeking help for their problems, compared to just 5–7% among the less severe procrastinators. There were also statistically significant differences with regard to the negative impact of procrastination on different life domains between the two groups, especially work/studies, *d* = 1.20–1.23.

**TABLE 2:** Differentiating severe procrastination from less severe procrastination.

		Less severe procrastination			Severe procrastination					
		*N*	Yes	In %	*N*	Yes	In %	Pearson Chi^2^	*df*	*p*
Female	PPS	344	233	68	385	255	66	0.18	1	0.67
	PDC	396	283	71	333	205	62	8.02	1	0.0046
Presently has a diagnosed psychiatric disorder	PPS	344	26	8	388	75	19	21.24	1	0.000
	PDC	398	43	11	334	58	17	6.57	1	0.0104
Considers procrastination a problem	PPS	344	143	42	388	374	96	261.17	1	0.000
	PDC	398	193	48	334	324	97	206.03		0.000
Has considered seeking help	PPS	344	18	5	388	134	35	26.92	1	0.000
	PDC	398	26	7	334	126	38	37.65		0.000
Meets procrastination criteria	PPS	344	48	14	388	286	74	262.46	1	0.000
GAD-7 score < 5 (no anxiety)	PPS	344	162	47	388	88	23	61.39	3	0.000
	PDC	398	182	46	334	68	20	73.9	3	0.000
GAD-7 score ≥ 5 (mild anxiety)	PPS	344	104	30	388	128	33			
	PDC	398	126	32	334	106	32			
GAD-7 score ≥ 10 (moderate anxiety)	PPS	344	51	15	388	87	22			
	PDC	398	59	15	334	79	24			
GAD-7 score ≥ 15 (severe anxiety)	PPS	344	27	8	388	85	22			
	PDC	398	31	8	334	81	24			
PHQ-9 score < 5 (no depression)	PPS	344	155	45	388	60	15	111.86	4	0.000
	PDC	398	173	43	334	42	13	122.84	4	0.000
PHQ-9 score ≥ 5 (mild depression)	PPS	344	111	32	388	109	28			
	PDC	398	125	31	334	95	28			
PHQ-9 score ≥ 10 (moderate depression)	PPS	344	48	14	388	98	25			
	PDC	398	64	16	334	82	25			
PHQ-9 score ≥ 15 (moderately severe depression)	PPS	344	19	6	388	71	18			
	PDC	398	22	6	334	68	20			
PHQ-9 score ≥ 20 (severe depression)	PPS	344	11	3	388	50	13			
	PDC	398	14	4	334	47	14			

GAD, Generalized Anxiety Disorder Questionnaire; PHQ, Patient Health Questionnaire. *p < 0.007 (Bonferroni correction).

**TABLE 3:** Differentiating severe procrastination from less severe procrastination.

			Less severe procrastination			Severe procrastination						
			*N*	*M*	*SD*	*N*	*M*	*SD*	t	df	p	d
Age		PPS	344	30.03	9.35	388	27.76	7.08	3.66	635.02	0.00*	0.28
		PDC	398	29.94	9.03	334	27.51	7.12	4.07	727.18	0.00*	0.30
Education credits		PPS	343	215.38	151.25	384	176.78	128.35	3.72	725	0.00*	0.28
		PDC	396	218.61	142.60	331	166.74	133.55	5.03	725	0.00*	0.37
Age start procrastination		PPS	143	18.44	7.15	374	16.45	5.51	3.00	209.60	0.00	0.33
		PDC	193	17.90	6.79	324	16.47	5.53	2.60	515	0.01	0.24
Effect of procrastination in life-domain												
	Interests/leisure	PPS	143	3.48	2.70	374	5.13	2.90	5.89	515	0.00*	0.58
		PDC	193	3.95	2.77	324	5.10	2.96	4.38	515	0.00*	0.40
	Work/studies	PPS	143	6.26	1.96	374	8.33	1.57	11.36	215.56	0.00*	1.23
		PDC	193	6.51	1.92	324	8.51	1.49	12.39	328.47	0.00*	1.20
	Friendships/social life	PPS	143	3.24	2.56	374	4.99	2.74	6.61	515	0.00*	0.65
		PDC	193	3.72	2.61	324	4.97	2.81	5.02	515	0.00*	0.46
	Community/engagement/Spirituality	PPS	143	2.08	2.57	374	3.48	3.32	5.05	329.58	0.00*	0.44
		PDC	193	2.53	2.90	324	3.42	3.31	3.19	445.93	0.00*	0.28
	Family life/parenting	PPS	143	2.83	2.44	374	4.05	2.87	4.87	300.63	0.00*	0.45
		PDC	193	3.05	2.64	324	4.11	2.84	4.28	426.73	0.00*	0.38
	Rest/sleep	PPS	143	5.27	2.98	374	6.77	2.89	5.23	515	0.00*	0.51
		PDC	193	5.62	3.03	324	6.80	2.88	4.43	515	0.00*	0.40
	Love/intimate relationships	PPS	143	2.97	2.84	374	4.10	3.25	3.91	292.19	0.00*	0.36
		PDC	193	3.24	3.10	324	4.11	3.18	3.03	515	0.00	0.28
	Physical activity/diet	PPS	143	4.78	2.76	374	6.50	2.86	6.29	265.52	0.00*	0.61
		PDC	193	5.21	2.99	324	6.50	2.79	4.87	381.28	0.00*	0.45

*p < 0.002 (Bonferroni correction)

**TABLE 4:** Differentiating severe procrastination from less severe procrastination.

		Less severe procrastination			Severe procrastination						
			
		*N*	*M*	*SD*	*N*	*M*	*SD*	*t*	*df*	*p*	*d*
Pure Procrastination Scale	PPS	344	2.20	0.53	388	3.71	0.50	39.43	730	0.00*	2.92
	PDC	398	2.46	0.75	334	3.65	0.62	23.58	729.93	0.00*	1.72
Susceptibility to Temptation Scale	PPS	344	2.60	0.81	388	3.64	0.73	18.20	695.24	0.00*	1.36
	PDC	398	2.70	0.85	334	3.68	0.72	16.97	729.95	0.00*	1.24
Clinical Perfectionism Questionnaire (Personal Standards)	PPS	344	2.73	0.63	388	2.60	0.62	2.71	730	0.01	0.20
	PDC	398	2.70	0.61	334	2.61	0.65	1.98	730	0.05	0.15
Clinical Perfectionism (Emotional Concerns)	PPS	344	2.64	0.74	388	3.19	0.68	10.49	730	0.00*	0.78
	PDC	398	2.65	0.75	334	3.26	0.62	12.05	729.99	0.00*	0.88
Generalized Anxiety Disorder Questionnaire	PPS	344	6.08	4.96	388	9.42	5.66	8.53	729.90	0.00*	0.63
	PDC	398	6.17	4.92	334	9.85	5.69	9.25	662.48	0.00*	0.70
Patient Health Questionnaire	PPS	344	6.33	5.36	388	11.28	6.24	11.53	729.28	0.00*	0.85
	PDC	398	6.65	5.52	334	11.70	6.15	11.59	676.20	0.00*	0.87
Perceived Stress Scale	PPS	344	2.59	0.58	388	3.19	0.57	–14.28	730	0.00*	1.06
	PDC	398	2.65	0.59	334	3.22	0.58	–13.08	730	0.00*	0.97
Brunnsviken Brief Quality of Life Scale: Leisure	PPS	344	8.74	4.72	388	7.10	4.75	4.68	730	0.00*	0.35
	PDC	398	8.71	4.60	334	6.87	4.86	5.25	730	0.00*	0.39
Brunnsviken Brief Quality of Life Scale: View of Life	PPS	344	10.78	4.80	388	8.72	5.23	5.52	730	0.00*	0.41
	PDC	398	10.59	4.80	334	8.61	5.33	5.23	677.10	0.00*	0.39
Brunnsviken Brief Quality of Life Scale: Creativity	PPS	344	7.49	4.96	388	6.24	5.19	3.32	730	0.00*	0.25
	PDC	398	10.59	4.80	334	8.61	5.33	5.23	677.10	0.00*	0.39
Brunnsviken Brief Quality of Life Scale: Learning	PPS	344	10.83	4.64	388	8.53	5.11	6.35	7300	0.00*	0.47
	PDC	398	7.47	4.87	334	6.07	5.31	3.74	730	0.00*	0.28
Brunnsviken Brief Quality of Life Scale: Friends	PPS	344	10.69	5.07	388	8.93	5.41	4.52	730	0.00*	0.45
	PDC	398	10.56	5.06	334	8.81	5.47	4.49	730	0.00*	0.33
Brunnsviken Brief Quality of Life Scale: Self	PPS	344	10.29	4.71	388	7.29	4.90	8.42	730	0.00*	0.62
	PDC	398	9.96	4.70	334	7.20	5.02	7.65	730	0.00*	0.57
Brunnsviken Brief Quality of Life Scale: Total	PPS	344	58.83	19.83	388	46.82	20.81	7.97	730	0.00*	0.59
	PDC	398	58.11	19.09	334	45.74	21.66	8.12	669.99	0.00*	0.61

*p < 0.002 (Bonferroni correction).

With the exception of perfectionism scores, severe cases and less severe cases of procrastination differed on all of the self-report measures, with severe procrastinators scoring higher on all measures and lower on quality of life. Moreover, the participants in the group “severe procrastination” also had a higher proportion of psychiatric disorders, and met the criteria for moderate and severe anxiety, and moderate and severe depression. From a demographic perspective, participants with severe procrastination were generally older and had achieved fewer higher education credits. When using the PPS to differentiate the groups, there were no gender differences. However, based on the PDC, the portion of female participants with severe procrastination was significantly lower than the portion of females in the group of less severe procrastination.

### Differential Overlap

Based on a median split on the PPS, 53% of the participants were considered to be severe procrastinators while applying the PDC, 46% of the participants were regarded as severe procrastinators. Combining the two revealed that among those being classified as severe procrastinators on the PPS, 74% were also identified as such based on the criteria of the PDC. Likewise, 86% of the participants being severe procrastinators on the PDC were recognized as such on the PPS. Overall, there was an overlap of 80% between the two methods for differentiating severe procrastination from less severe procrastination. Also, the 20% non-overlap was not equally distributed between the severe cases (32% of non-overlap), and less severe cases (68% of non-overlap) of procrastination. In other words, both ways might be reliable in identifying cases of severe procrastination, but the PPS could potentially overreport the number of severe cases. Furthermore, the PDC might be more sensitive to gender differences as it demonstrates that the proportion of female participants in the group “severe procrastination” is lower than the proportion of female non-severe procrastinators.

### Physical and Psychological Issues of Procrastination

#### Physical Issues

The participants reported a large number of physical issues that are considered emblematic of *Stress and anxiety*, see Table 5 for an overview. These could in turn be organized according to six subthemes; *Tension* (e.g., feeling tensed around your shoulders, neck, and back), *Pain* (e.g., bruxism, muscular pain, and experiencing recurrent headaches or migraine), *Sickness* (e.g., nausea, dizziness, and shudders), *Stomach* (e.g., increased or decreased appetite, stomach aches, and diarrhea), and *Sleep and rest* (e.g., insomnia, tiredness, and restlessness). In a majority of the cases, participants described having more than one symptom, such as feeling stressed out, having difficulties sleeping, and being restless.

**TABLE 5:** Physical issues of procrastination.

Theme	Subthemes	Characteristics	Example
Stress and anxiety			
	Tension	Feeling tensed around your shoulders, neck, and back	*“Physical signs of anxiety with headaches, tensions in my neck and shoulders, and difficulties relaxing.”*
	Pain	Bruxism, muscular pain, headaches, and migraine	*“Pain in my back and neck when I have to study long hours before an exam, which I’ve procrastinated.”*
	Sickness	Nausea, dizziness, and shudders	*“Dizziness, inertia, and brain fog.”*
	Stomach	Increased or decreased appetite, stomach aches, and diarrhea	*“Lack of appetite, followed by periods of overindulgence.”*
	Sleep and rest	Insomnia, tiredness, and restlessness	*“Difficulties sleeping. I wake up at night several times and am unable to go back to sleep.”*
	Other	Worsening of an underlying condition (e.g., eczema or irritable bowel syndrome) or other symptoms (e.g., biting your nails)	*“Mostly stomach issues. I was diagnosed with IBS* [irritable bowel syndrome] *when I was 17. Always get an upset stomach when I’m stressed out over tasks or when I’m stressed out in general.”*

Among the less common physical issues, *Other*, these were characterized by the worsening of an already underlying condition, such as eczema, causing flare ups or exacerbated problems. However, a few participants also mentioned biting their nails when under stress or experiencing problems with gastritis or becoming numb.

#### Psychological Issues

In terms of the psychological issues, there was a clear overlap with many of the physical symptoms described above, see Table 6 for an overview. One of the overarching themes, *Stress and anxiety*, included four subthemes; *Sleep and rest* (e.g., insomnia, tiredness, restlessness, and feeling exhausted), *Fear* (e.g., worrying about your current situation or the future and feelings of panic), *Cognitive load* (e.g., having difficulties concentrating and remembering things), and *Performance* (e.g., experiencing performance anxiety or having difficulties achieving high standards).

**TABLE 6:** Psychological issues of procrastination.

Theme	Subthemes	Characteristics	Example
Stress and anxiety			
	Sleep and rest	Insomnia, tiredness, restlessness and exhaustion	*“Difficulties sleeping, particularly with regard to falling asleep, but also waking up at night, often between 3 and 4 a.m.”*
	Fear	Worry and panic	*“Anxious about falling behind, not being able to succeed on my exams.”*
	Cognitive load	Difficulties concentrating and remembering things	*“Anxiety, worry, difficulties concentrating, and problems remembering things.”*
	Performance	Performance anxiety and difficulties achieving high standards	*“Anxiety preceding tests and exams that I wasn’t able to commit study hours toward.”*
Depression	Self-criticism	Self-loathing, feelings of disappointment, and negative thoughts	*“Self-loathing (pushing myself down, feel incapable of doing things, etc. Constantly comparing myself to others.”*
	Remorse	Anger, frustration, and shame	*“Anxiety, remorse, and shame that I continue to procrastinate even though I know it’s stupid.”*
	Self-esteem	Feeling inadequate and loss of self-confidence	*“Anxiety, disappointment, stress, fear, lower self-esteem, inadequate, not being smart enough, and that you’re not worthwhile.”*
Other	Other	Eating disorders, compulsions, and social anxiety	*“Panic, a mild form of self-harm, a general sense of disappointment which affects the way I perceive myself.”*

Apart from being stressed out and anxious, most participants also described having a lower mood, and feelings of hopelessness and despair. This overarching theme, *Depression*, consisted of three subthemes; *Self-criticism* (e.g., self-loathing, feelings of disappointment with oneself, and negative thoughts), *Remorse* (e.g., anger, frustration, and feelings of shame), and *Self-esteem* (e.g., feeling inadequate and experiencing a loss of self-confidence).

Less prevalent were signs of *Other* conditions and symptoms, such as eating disorders, compulsions, and social anxiety, although a few participants experienced these issues in relation to their procrastination.

## Discussion

### General Discussion

The first aim of the current study was to explore ways of differentiating students who might require professional help for procrastination from those with less pressing matters. Overall, the findings suggest that cases of severe procrastination, as determined using either the PPS or the PDC, are characterized by higher levels of anxiety, depression, and stress than the less severe cases, representing moderate to large between-group effect sizes. Given the magnitude of these differences, severe procrastinators could therefore warrant further assessment and possibly even treatment, such as via a student health center. Furthermore, severe procrastination was associated with greater self-reported negative effects on all of the life-domains that were examined, most notably for work/studies, but also for physical activity/diet and rest/sleep, which resemble previous research on the impact of procrastination on both academic achievement and health (e.g., Grunschel et al., 2013; Kim and Seo, 2015). In addition, quality of life was more negatively affected among severe procrastinators, corresponding to moderate between-group effect sizes, although, the level of quality of life was not as impaired as has been found in clinical samples (Lindner et al., 2016). As for impulsivity, those with severe procrastination were far more susceptible to temptation, a difference consistent with a large between-group effect sizes, which is in line with the idea of impulsivity being one of the strongest personality traits predictive of procrastination (Steel, 2007). With regard to perfectionism, only emotional concerns differed between severe and less severe procrastinators, corresponding to large between-group effect sizes. Similar to the findings by Sirois et al. (2017), emotional, or, neurotic, aspects of perfectionism thus appear to be much more strongly related to severe procrastination, suggesting that students who are concerned about making mistakes and not living up to certain standards might need treatment that specifically target these issues.

When explicitly asked about it, severe procrastinators seem to regard procrastination as a problem to a much greater extent than less severe procrastinators (96 and 97%, in comparison to 42 and 48%, depending on whether the PPS or the PDC was used for differentiation), something they also report having been more inclined to seek help for (35 and 38% compared to 5 and 7%). This is the first time such direct queries have been used to determine if someone might need further assistance, giving some credence to the results and pointing toward the utility of using either the PPS or the PDC to identify severe cases of procrastination. However, as indicated in the current study, the PPS could potentially overreport the number of severe cases. Meanwhile, the PDC might be more sensitive to gender differences as it demonstrates that the proportion of female participants among the severe procrastinators is significantly lower than the proportion of female participants among the less severe procrastinators.

Another aim of the current study was to understand the physical and psychological issues related to procrastination by investigating the responses to two open-ended items. In terms of the former, the results demonstrate that many students who procrastinate experience symptoms that are commonly seen in stress and anxiety, such as being tensed, having sleeping problems, and struggling with different forms of pain. These issues are in line with the findings by Grunschel et al. (2013) who also reported a high incidence of such consequences from procrastinating. In addition, it corroborates the procrastination-health model by Sirois (2007), which proposed that stress might act as a mediator between procrastination and many physical issues. The idea that procrastination is associated with stress, and, in turn, leads to other concerns, is reasonable given the nature of procrastination. While it may decrease discomfort temporarily (cf. Sirois and Pychyl, 2013), the activity being postponed still has to be performed on a later occasion, causing more stress overall (Tice and Baumeister, 1997).

As for the psychological issues, these were also characterized by symptoms of stress and anxiety, for example, insomnia, restlessness, and worry, suggesting a high degree of overlap with the physical issues. Again, this corresponds to the results by Grunschel et al. (2013), and should be seen as the affective and somatic effects of being anxious and stressed out from procrastinating. Furthermore, difficulties concentrating and remembering things are not uncommon when under stress, thereby affecting the possibility to pursue a given action (Marin et al., 2011), as reported by many participants in the current study. However, a noticeable difference between the physical and psychological issues are aspects related to performance, self-criticism, remorse, and self-esteem. These might portray the more depressogenic impact of procrastination, such as being disappointed with oneself, experiencing lower self-confidence, and exhibiting negative self-evaluation. This goes in line with the notion of efficacy-performance spirals, whereby the inability to execute goal-directed behaviors and progress toward a given end-point can lead to lower mood, self-loathing, and decreased motivation (Lindsley et al., 1995). In other words, procrastination does not only appear to cause stress and anxiety in the aftermath of a procrastination episode, but also negatively impacts the general state of the individual by inducing self-doubt, frustration, shame, rumination, and feelings of inadequacy (cf. Giguère et al., 2016; Constantin et al., 2018). When demonstrating such depressive thoughts and feelings, it is then not unreasonable to expect the person to be less inclined to take care of the assignments that need to be done, further perpetuating a downward cycle.

### Practical Implications and Recommendations

Based on the results from the current study, the PPS is recommended as an initial screening tool for large samples, such as when admitting new students to a study program or as a general assessment of well-being at a university. As a second step, students who score higher than a certain cut-off (e.g., 3.00 like in the present study) on the items should be advised to fill out the PDC to more accurately determine the severity level of procrastination and its associated physical and psychological issues. This procedure could, for instance, be implemented at a student health center in order to identify those students in need of professional help, although it should be noted that the PDC has so far only been used in this way in Germany. In addition, administering the GAD-7 and PHQ-9 on the same occasion gives some indication of symptoms of anxiety and depression. This would inform therapists of other possible conditions that might warrant their attention, such as major depressive disorder, which sometimes have to be dealt with first in treatment. Furthermore, for those who seek support for procrastination, discussing the criteria of the PDC and the physical and psychological issues presented in the current study might help them understand what they are experiencing and how to overcome their problems. This type of psychoeducation can often have a normalizing effect, reducing shame and stigma, and, in turn, motivate behavior change. Similarly, career counselors might use the PDC in relation to discussing study satisfaction and dropout intentions in order to prevent students ending their studies prematurely (Scheunemann et al., 2021).

Apart from aiding the identification of severe procrastinators, the findings from the current study may also have implications for treatment. The physical and psychological issues reported by the participants suggest that symptoms of stress and anxiety are common. On the one hand, procrastination can sometimes be a response to this discomfort. On the other hand, procrastinating an activity can also give rise to this distress (Rozental and Carlbring, 2014). In both cases, interventions targeting symptoms of stress and anxiety seem important in order to overcome many difficulties experienced by students, which can involve goal-setting, problem-solving, time management, and exposure to negative emotions, as have been tested in clinical trials (e.g., Rozental et al., 2015, 2018). The basic tenet is to lower stress levels and help endure those feelings that might otherwise lead one astray. Moreover, the depressogenic impact of procrastination may cause the individual to feel less willing to initiate goal-directed behaviors. Similar to the actions of someone suffering from major depressive disorder, this however, prevents the person from experiencing mastery and joy, furthering a vicious process of passivity and negative self-evaluation. Interventions that focus on activity scheduling and step-wise performance of activities might therefore be key to overcoming inaction and self-loathing, i.e., behavioral activation (Ramsay, 2002). Likewise, students who may be experiencing low self-efficacy due to their procrastination could benefit from study skills training (Svartdal et al., 2021). Concerning the different phases of a procrastination episodes (Svartdal et al., 2020b), it might even be worthwhile to differentiate between strategies that upregulate motivation as in motivational regulation strategies (Grunschel et al., 2016), and strategies that downregulate negative affect (Eckert et al., 2016), thus, tailoring them to the specific needs of the student. Furthermore, the environment for many students also seems to result in procrastination and might have to be targeted. Svartdal et al. (2020a) provide an overview of the measures that could be taken by course coordinators and lecturers, such as study skills training, group work, and courses in self-regulation.

### Limitations

The current study is, to the knowledge of the authors, the first attempt at differentiating the more severe from less severe procrastinators among university students. It has furthered the understanding of what characterizes problematic forms of procrastination and provided recommendations on how to screen and support those experiencing difficulties completing their commitments. However, there are also several limitations that need to be addressed.

First, recruitment of participants was made via advertisements and information distributed universities and in relevant forums. Although a reasonable way of reaching university students, it might also have attracted proportionally more individuals with greater problems of procrastination or, the other way around, those for whom procrastination is just a little nuisance. This self-selection bias might have affected the possibility to differentiate between “severe procrastination” and “less severe procrastination.” The distribution of scores on the self-report measures do not seem to suggest that this is the case, but future research should try alternative methods of recruiting participants, such as stratified random sampling. Similarly, the current study focused on students in university settings only, making it unclear whether the results can be generalized to an adult working population or younger students in elementary school or high-school. Replicating the approach used here should be feasible in other settings in order to determine if the same type of classification is possible to make elsewhere. Replicating the approach in a longitudinal design would, furthermore, deliver information on causal relationships between procrastination and psychopathological symptoms.

Second, the current study was conducted during the fall semester of 2020, which is about 6 months into the COVID-19 pandemic. Similar to other countries, universities in Sweden shut down on-campus education during the spring of the same year, meaning that most curricular activity was performed online when the participants responded to the survey. Whether this has affected university students’ levels of procrastination is not known, but given the lack of routines and social support it is reasonable to assume that it has been detrimental to some. Furthermore, the COVID-19 pandemic itself, and its effects of everyday life, might have affected the physical and psychological well-being of some participants, thereby inflating the scores of the self-report measures somewhat.

Third, we used a median split on the PPS for differentiating the more severe from less severe procrastinators. In general, median splits, as practice for dichotomizing a continuous variable, have a long tradition of being criticized for the loss of information and reduction in power (e.g., Cohen, 1983). However, newer studies weaken this criticism considerably (e.g., Iacobucci et al., 2015a,b) by showing that this is in fact a robust method. For our purpose, it was very important to retain all information of the sample. Splitting the sample into three groups and only using the two extreme one would have resulted in a considerable loss of information, albeit useful for therapists. The median split of the PPS, however, and the diagnostic criteria used in the PDC, have not previously been tested regarding their classification accuracy for identifying more severe procrastinators. It is therefore unknown if these two methods can be applied for this purpose. Usually, a gold standard is used for comparison and validation, such as a structured clinical interview for determining major depressive disorder. However, such a diagnostic procedure is not possible for procrastination because it is not considered to be a diagnosis. Instead, the current study asked questions on whether the participants themselves regarded procrastination as a problem and if they ever considered seeking help for procrastination as a proxy for diagnosis. An idea for future research is to corroborate this method by interviews, which may provide additional insights on where to place the cutoff between severe and less severe procrastination.

## Data Availability Statement

The raw data supporting the conclusions of this article will be made available by the authors, without undue reservation.

## Ethics Statement

The studies involving human participants were reviewed and approved by Swedish Ethical Review Authority (Dnr: 2020-00555). The patients/participants provided their online informed consent to participate in this study.

## Author Contributions

AR and KK designed the study and outlined its research aims and drafted the manuscript. AR and DF applied for ethics approval, set up the study, and monitored the data collection. AH advertised the study and managed the recruitment of participants. KK was responsible for the quantitative analyses. AR and DF was responsible for the qualitative analyses. DF and AH commented on the manuscript and approved its submission. All the authors contributed to the article and approved the submitted version.

## Conflict of Interest

The authors declare that the research was conducted in the absence of any commercial or financial relationships that could be construed as a potential conflict of interest. The reviewer TD declared a past co-authorship with one of the authors KK to the handling editor. The handling editor declared a past co-authorship with one of the authors KK.

## Publisher’s Note

All claims expressed in this article are solely those of the authors and do not necessarily represent those of their affiliated organizations, or those of the publisher, the editors and the reviewers. Any product that may be evaluated in this article, or claim that may be made by its manufacturer, is not guaranteed or endorsed by the publisher.

## Appendix I

**Table:** Comparing completers with non-completers.

	Completers (*N* = 732)		Non-completers					
	*M*	*SD*	*M*	*SD*	*N*	*t*	*df*	*p*
Age	28.83	8.30	28.82	6.85	65	0.12	795	0.99
Achieved education credits	194.99	140.85	174.14	113.90	64	1.15	789	0.25
Pure Procrastination Scale	3.00	0.91	2.98	1.00	13	0.08	743	0.94
Gender identity	67% female		70% female		64			
Civil status	44% single		56% single		63			
	54% married		40% married					
	2% divorced		5% divorced					
Children	At home 20%		At home 20%		65			
	Not at home 2%		Not at home 1%					
	No 78%		No 79%					

## Appendix II

**Table:** Descriptive statistics and correlations for the whole sample (*N* = 732).

		*M*	*SD*	2	3	4	5	6	7	8	9	10	11	12	13	14	15	16
1	Age	28.83	8.30	0.38**	−0.16**	−0.23**	0.04**	−0.17**	−0.16**	−0.20**	−0.17**	0.01**	0.11**	0.10**	0.24**	−0.02**	0.06**	−0.21**
2	Age_problematic	17.00	6.07		−0.18**	−0.21**	0.06**	−0.04**	0.03**	−0.02**	−0.01**	−0.11**	0.01**	−0.07**	0.06**	−0.03**	−0.03**	−0.22**
3	PPS	3.00	0.91			0.71**	−0.10**	0.43**	0.39**	0.48**	0.57**	−0.23**	−0.27**	−0.16**	−0.31**	−0.21**	−0.36**	−0.06**
4	STS	3.15	0.93				−0.13**	0.36**	0.30**	0.38**	0.44**	−0.10**	−0.22**	−0.07**	−0.27**	−0.12**	−0.30**	−0.24**
5	CPQ_Personal Standards	2.66	0.63					0.35**	0.31**	0.20**	0.17**	−0.20**	0.01**	0.02**	0.08**	−0.01**	−0.09**	0.20**
6	CPQ_Emotional Concerns	2.93	0.76						0.55**	0.55**	0.60**	−0.31**	−0.26**	−0.19**	−0.30**	−0.24**	−0.46**	0.07**
7	GAD	7.85	5.59							0.76**	0.70**	−0.43**	−0.33**	−0.24**	−0.31**	−0.29**	−0.45**	−0.17**
8	PHQ	8.95	6.34								0.72**	−0.45**	−0.40**	−0.26**	−0.40**	−0.34**	−0.55**	−0.30**
9	PSS	2.91	0.65									−0.48**	−0.38**	−0.30**	−0.42**	−0.34**	−0.54**	−0.50**
10	BBQ_Leisure	7.87	4.81										0.40**	0.40**	0.33**	0.43**	0.41**	0.39**
11	BBQ_View of Life	9.68	5.14											0.42**	0.41**	0.38**	0.53**	0.24**
12	BBQ_Creativity	6.83	5.12												0.40**	0.28**	0.30**	0.54**
13	BBQ_Learning	9.61	5.03													0.29**	0.40**	0.48**
14	BBQ_Friends	9.76	5.32														0.38**	0.35**
15	BBQ_Self	8.70	5.04															0.23**
16	BBQ_Total	52.46	21.21															

*Age_problematic, age procrastination started to be perceived as problematic; PPS, Pure Procrastination Scale; STS, Susceptibility to Temptation Scale; CPQ, Clinical Perfectionism Scale; GAD, General Anxiety Scale; PHQ, Patient Health Questionnaire; PSS, Perceived Stress Scale; BBQ, Brunnsviken Brief Quality of Life Scale. *p ≤ 0.5, **p ≤ 0.1.*

## References

[B1] American Psychiatric Association (2013). *Diagnostic and Statistical Manual of Mental Disorders.* Washington, DC: American Psychiatric Association. 10.1176/appi.books.9780890425596

[B2] Beutel M. E. Klein E. M. Aufenanger S. Brähler E. Dreier M. Müller K. W. (2016). Procrastination, distress and life satisfaction across the age range – a german representative community study. *PLoS One* 11:e0148054. 10.1371/journal.pone.0148054 26871572 PMC4752450

[B3] Braun V. Clark V. (2006). Using thematic analysis in psychology. *Qualit. Res. Psychol.* 3 77–101. 10.1191/1478088706qp063oa 32100154

[B4] Buhrman M. Gelberg O. Jovicic F. Molin K. Forsström D. Andersson G. (2020). Treating perfectionism using internet-based cognitive behavior therapy: a study protocol for a randomized controlled trial comparing two types of treatment. *Int. Intervent.* 21:100338. 10.1016/j.invent.2020.100338 32944504 PMC7481556

[B5] Cohen J. (1983). The cost of dichotomization. *Appl. Psychol. Measure.* 7 249–253. 10.1177/014662168300700301

[B6] Cohen S. Kamarck T. Mermelstein R. (1983). A global measure of perceived stress. *J. Health Soc. Behav.* 24 386–396. 10.2307/2136404 6668417

[B7] Constantin K. English M. M. Mazmanian D. (2018). Anxiety, depression, and procrastination among students: rumination plays a larger mediating role than worry. *J. Rational Emotive Cogn. Behav. Ther.* 36 15–27. 10.1007/s10942-017-0271-5

[B8] Day V. Mensink D. O’Sullivan M. (2000). Patterns of academic procrastination. *J. College Reading Learn.* 30 120–134. 10.1080/10790195.2000.10850090

[B9] Dickie L. Surgenor L. J. Wilson M. McDowall J. (2012). The structure and reliability of the clinical perfectionism questionnaire. *Personal. Indiv. Diff.* 52 865–869. 10.1016/j.paid.2012.02.003

[B10] Eckert M. Ebert D. D. Lehr D. Sieland B. Berking M. (2016). Overcome procrastination: enhancing emotion regulation skills reduce procrastination. *Learn. Indiv. Diff.* 52 10–18. 10.1016/j.lindif.2016.10.001

[B11] Ehlers A. Wild J. Warnock-Parkes E. Grey N. Murray H. Kerr A. (2020). A randomised controlled trial of therapist-assisted online psychological therapies for posttraumatic stress disorder (STOP-PTSD): trial protocol. *Trials* 21 1–13. 10.1186/s13063-020-4176-8 32326954 PMC7181498

[B12] Engberding M. Frings E. Höcker A. Wolf J. Rist F. (2011). “Is procrastination a symptom or a disorder like other axis-1-disorders in the DSM? Steps towards delineating a case definition,” in *Proceeding of the Presentation at the 7th Biennal Conference on Procrastiantion*, (Amsterdam).

[B13] Giguère B. Sirois F. M. Vaswani M. (2016). “Delaying things and feeling bad about it? A norm-based approach to procrastination,” in *Procrastination, Health, and Well-Being*, eds Sirois F. M. Pychyl T. A. (Academic Press), 189–212. 10.1016/B978-0-12-802862-9.00009-8

[B14] Grunschel C. Schopenhauer L. (2015). Why are students (not) motivated to change academic procrastination? An investigation based on the transtheoretical model of change. *J. College Stud. Dev.* 56 187–200. 10.1353/csd.2015.0012 34409987

[B15] Grunschel C. Patrzek J. Fries S. (2013). Exploring reasons and consequences of academic procrastination: an interview study. *Eur. J. Psychol. Educ.* 28 841–861. 10.1007/s10212-012-0143-4

[B16] Grunschel C. Schwinger M. Steinmayr R. Fries S. (2016). Effects of using motivational regulation strategies on students’ academic procrastination, academic performance, and well-being. *Learn. Indiv. Diff.* 49 162–170. 10.1016/j.lindif.2016.06.008

[B17] Gustavson D. E. Miyake A. Hewitt J. K. Friedman N. P. (2014). Genetic relations among procrastination, impulsivity, and goal-management ability: implications for the evolutionary origin of procrastination. *Psychol. Sci.* 25 1178–1188. 10.1177/0956797614526260 24705635 PMC4185275

[B18] Harriot J. Ferrari J. R. (1996). Prevalence of procrastination among samples of adults. *Psychol. Rep.* 78 611–616. 10.2466/pr0.1996.78.2.611

[B19] Höcker A. Engberding M. Rist F. (2017). *Prokrastination – Ein Manual Zur Behandlung Des pathologischen Aufschiebens [Procrastination - A Manual for the Treatment of Pathological Delay].* Germany: Hogrefe.

[B20] Iacobucci D. Posavac S. S. Kardes F. R. Schneider M. J. Popovich D. L. (2015a). Toward a more nuanced understanding of the statistical properties of a median split. *J. Consumer Psychol.* 25 652–665. 10.1016/j.jcps.2014.12.002

[B21] Iacobucci D. Posavac S. S. Kardes F. R. Schneider M. J. Popovich D. L. (2015b). The median split: robust, refined, and revived. *J. Consumer Psychol.* 25 690–704. 10.1016/j.jcps.2015.06.014

[B22] Kim K. R. Seo E. H. (2015). The relationship between procrastination and academic performance: a meta-analysis. *Personal. Indiv. Diff.* 82 26–33. 10.1016/j.paid.2015.02.038

[B23] Klingsieck K. B. (2013). Procrastination: when good things don’t come to those who wait. *Eur. Psychol.* 18 24–34. 10.1027/1016-9040/a000138

[B24] Klingsieck K. B. Grund A. Schmid S. Fries S. (2013). Why students procrastinate: a qualitative approach. *J. College Stud. Dev.* 54 397–412. 10.1353/csd.2013.0060 34409987

[B25] Kroenke K. Spitzer R. L. Williams J. B. (2001). The PHQ−9: validity of a brief depression severity measure. *J. General Int. Med.* 16 606–613. 10.1046/j.1525-1497.2001.016009606.x 11556941 PMC1495268

[B26] Lannoy S. Billieux J. Poncin M. Maurage P. (2017). Binging at the campus: motivations and impulsivity influence binge drinking profiles in university students. *Psychiatry Res.* 250 146–154. 10.1016/j.psychres.2017.01.068 28161610

[B27] Lindner P. Frykheden O. Forsström D. Andersson E. Ljótsson B. Hedman E. (2016). The brunnsviken brief quality of life scale (BBQ): development and psychometric evaluation. *Cogn. Behav. Ther.* 45 182–195. 10.1080/16506073.2016.1143526 26886248 PMC4867878

[B28] Lindsley D. H. Brass D. J. Thomas J. B. (1995). Efficacy-performing spirals: a multilevel perspective. *Acad. Manage. Rev.* 20 645–678. 10.5465/amr.1995.9508080333

[B29] Loehlin J. C. Martin N. G. (2014). The genetic correlation between procrastination and impulsivity. *Twin Res. Hum. Genet.* 17 512–515. 10.1017/thg.2014.60 25431285

[B30] Marin M. F. Lord C. Andrews J. Juster R. P. Sindi S. Arsenault-Lapierre G. (2011). Chronic stress, cognitive functioning and mental health. *Neurobiol. Learn. Memory* 96 583–595. 10.1016/j.nlm.2011.02.016 21376129

[B31] Ramsay J. R. (2002). A cognitive therapy approach for treating chronic procrastination and avoidance: behavioral activation interventions. *J. Group Psychother. Psychodr. Soiometry* 55 79–93. 10.3200/JGPP.55.2.79-92

[B32] Reilly E. D. Ritzert T. R. Scoglio A. A. Mote J. Fukuda S. D. Ahern M. E. (2019). A systematic review of values measures in acceptance and commitment therapy research. *J. Context. Behav. Sci.* 12 290–304. 10.1016/j.jcbs.2018.10.004

[B33] Rozental A. Carlbring P. (2014). Understanding and treating procrastination: a review of a common self-regulatory failure. *Psychology* 5 1488–1502. 10.4236/psych.2014.513160

[B34] Rozental A. Forsell E. Svensson A. Forsström D. Andersson G. Carlbring P. (2014). Psychometric evaluation of the Swedish version of the pure procrastination scale, the irrational procrastination scale, and the susceptibility to temptation scale in a clinical population. *BMC Psychol.* 2:54. 10.1186/s40359-014-0054-z 25566392 PMC4269972

[B35] Rozental A. Forsell E. Svensson A. Andersson G. Carlbring P. (2015). Internet-based cognitive-behavior therapy for procrastination: a randomized controlled trial. *J. Consul. Clin. Psychol.* 83 808–824. 10.1037/ccp0000023 25939016

[B36] Rozental A. Forsström D. Lindner P. Nilsson S. Mårtensson L. Rizzo A. (2018). Treating procrastination using cognitive behavior therapy: a pragmatic randomized controlled trial comparing treatment delivered via the internet or in groups. *Behav. Ther.* 49 180–197. 10.1016/j.beth.2017.08.002 29530258

[B37] Scheunemann A. Schnettler T. Bobe J. Fries S. Grunschel C. (2021). A longitudinal analysis of the reciprocal relationship between academic procrastination, study satisfaction, and dropout intentions in higher education. *Eur. J. Psychol. Educ.* 1–24. 10.1007/s10212-021-00571-z [Epub ahead of print].

[B38] Sirois F. M. (2007). “I’ll look after my health, later”: a replication and extension of the procrastination–health model with community-dwelling adults. *Pers. Individ. Differ.* 43 15–26. 10.1016/j.paid.2006.11.003

[B39] Sirois F. M. Pychyl T. (2013). Procrastination and the priority of short−term mood regulation: consequences for future self. *Soc. Personal. Psychol. Compass* 7 115–127. 10.1111/spc3.12011

[B40] Sirois F. M. Melia-Gordon M. L. Pychyl T. A. (2003). “I’ll look after my health, later”: an investigation of procrastination and health. *Personal. Indiv. Diff.* 35 1167–1184. 10.1016/S0191-8869(02)00326-4

[B41] Sirois F. M. Molnar D. S. Hirsch J. K. Back M. (2017). A meta–analytic and conceptual update on the associations between procrastination and multidimensional perfectionism. *Eur. J. Personal.* 31 137–159. 10.1002/per.2098

[B42] Sirios F. Pychyl T. (2016). *Procrastination, Health, and Well-Being.* Cambridge, MA: Academic Press. 10.1016/B978-0-12-397045-9.00166-X

[B43] Solomon L. J. Rothblum E. D. (1984). Academic procrastination: frequency and cognitive-behavioral correlates. *J. Counsel. Psychol.* 31 503–509. 10.1037/0022-0167.31.4.503

[B44] Spitzer R. L. Kroenke K. Williams J. B. Löwe B. (2006). A brief measure for assessing generalized anxiety disorder: the GAD-7. *Arch. Int. Med.* 166 1092–1097. 10.1001/archinte.166.10.1092 16717171

[B45] Stead R. Shanahan M. J. Neufeld R. W. (2010). “I’ll go to therapy, eventually”: procrastination, stress and mental health. *Personal. Indiv. Diff.* 49 175–180. 10.1016/j.paid.2010.03.028

[B46] Steel P. (2007). The nature of procrastination: a meta-analytic and theoretical review of quintessential self-regulatory failure. *Psychol. Bull.* 133 65–94. 10.1037/0033-2909.133.1.65 17201571

[B47] Steel P. (2010). Arousal, avoidant and decisional procrastinators: do they exist? *Personal. Indiv. Diff.* 48 926–934. 10.1016/j.paid.2010.02.025

[B48] Steel P. Klingsieck K. B. (2016). Academic procrastination: psychological antecedents revisited. *Austr. Psychol.* 51 36–46. 10.1111/ap.12173

[B49] Stoeber J. Hotham S. (2013). Perfectionism and social desirability: students report increased perfectionism to create a positive impression. *Personal. Indiv. Diff.* 55 626–629. 10.1016/j.paid.2013.04.023

[B50] Svartdal F. Klingsieck K. B. Steel P. Gamst-Klaussen T. (2020b). Measuring implemental delay in procrastination: separating onset and sustained goal striving. *Personal. Indiv. Diff.* 156: 109762. 10.1016/j.paid.2019.109762 [Epub ahead of print].

[B51] Svartdal F. Dahl T. I. Gamst-Klaussen T. Koppenborg M. Klingsieck K. B. (2020a). How study environments foster academic procrastination: overview and recommendations. *Front. Psychol.* 11: 3005. 10.3389/fpsyg.2020.540910 33224046 PMC7667251

[B52] Svartdal F. Pfuhl G. Nordby K. Foschi G. Klingsieck K. B. Rozental A. (2016). On the measurement of procrastination: comparing two scales in six european countries. *Front. Psychol.* 7:1307. 10.3389/fpsyg.2016.01307 27630595 PMC5005418

[B53] Svartdal F. Sæle R. G. Dahl T. I. Nemtcan E. Gamst-Klaussen T. (2021). Study habits and procrastination: the role of academic self-efficacy. *Scand. J. Educ. Res.* 1–20. 10.1080/00313831.2021.1959393

[B54] Swedish Higher Education Authority (2020). *Fler studenter i högskolan 2019-2020.* Available online at: https://www.uka.se/om-oss/aktuellt/nyheter/2020-10-13-fler-studenter-i-hogskolan-2019-2020.html (accessed October 13, 2020)

[B55] Thomas D. R. (2006). A general inductive approach for analyzing qualitative evaluation data. *Am. J. Evalu.* 27 237–246. 10.1177/1098214005283748

[B56] Tice D. M. Baumeister R. F. (1997). Longitudinal study of procrastination, performance, stress, and health: the costs and benefits of dawdling. *Psychol. Sci.* 8 454–458. 10.1111/j.1467-9280.1997.tb00460.x

[B57] van Eerde W. (2003). A meta-analytically derived nomological network of procrastination. *Personal. Indiv. Diff.* 35 1401–1418. 10.1016/S0191-8869(02)00358-6

